# Review of recent advances in inorganic photoresists†

**DOI:** 10.1039/c9ra08977b

**Published:** 2020-02-28

**Authors:** Chaoyun Luo, Chanchan Xu, Le Lv, Hai Li, Xiaoxi Huang, Wei Liu

**Affiliations:** School of Applied Chemistry and Biological Technology, Shenzhen Polytechnic 7098 Liuxian Blvd, Nanshan District Shenzhen 518055 China; Hoffmann Institute of Advanced Materials, Shenzhen Polytechnic 7098 Liuxian Blvd, Nanshan District Shenzhen 518055 China xiaoxihuang@szpt.edu.cn weiliu2018@szpt.edu.cn; Postdoctoral Innovation Practice Base, Shenzhen Polytechnic 7098 Liuxian Blvd, Nanshan District Shenzhen 518055 China

## Abstract

The semiconductor industry has witnessed a continuous decrease in the size of logic, memory and other computer chip components since its birth over half a century ago. The shrinking of features has to a large extent been enabled by the development of advanced photolithographic techniques. This review focuses on one important component of lithography, the resist, which is essentially a thin film that can generate a specific feature after an exposure and development process. Smaller features require an even more precisely focused photon, electron or ion beam with which to expose the resist. The promising light source for next generation lithography that will enable downscaling patterns to be written is extreme ultraviolet radiation (EUV), 92 eV (13.5 nm). The review mainly focuses on inorganic resists, as they have several advantages compared with traditional organic resists. In order to satisfy the throughput requirement in high volume semiconductor manufacturing, metal oxide resists with high resolution and sensitivity have been proposed and developed for EUV lithography. The progress of various inorganic resists is introduced and their properties have been summarized.

## Abstract 2

The current review aims to focus on recent progress and opportunities in inorganic photoresist materials, including their fabrication process, performance and working mechanism.

## Graphical abstract

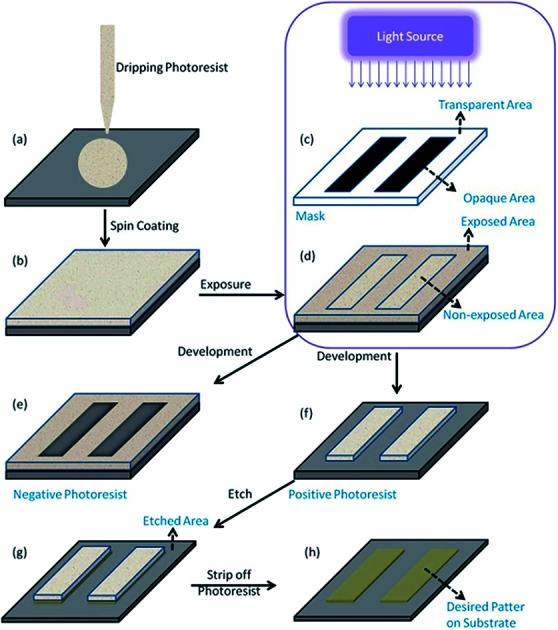

## 1. Introduction

**Fig. 1:**
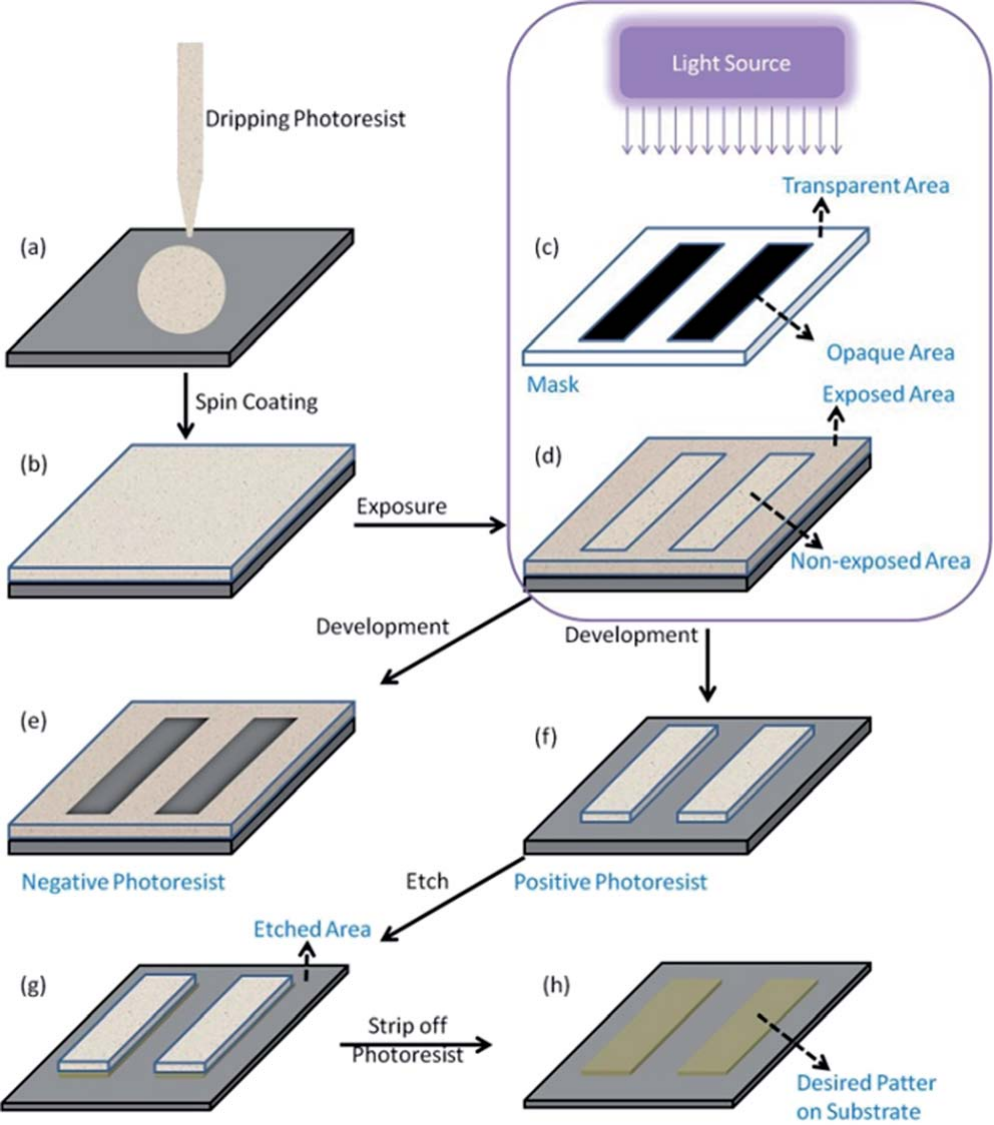
Schematic diagram of a photolithography technology process. (a) Apply photoresist on substrate surface; (b) prepare photoresist film *via* spin coating; (c) mask with transparent and opaque areas; (d) exposed photoresist film; (e) negative photoresist *via* negative tone development; (f) positive photoresist *via* positive tone development; (g) selective removal of substrate with the help of photoresist during etching process; (h) desired pattern on substrate after striping off photoresist.

With the increasing demand for portable electronic products, such as mobile phones, computers, portable medical equipment, *etc.*, micro–nano processing technology has received more and more attention in modern semiconductor manufacturing.^1,2^ The structural dimensions prepared by using this technology are in the micron to nano size range, such as high-performance integrated circuits, display panels, high-precision components, *etc.*^3^ Examples of micro-nano processing technologies are 3D printing technology, photolithography technology and nanoimprint technology. Photolithography refers to the technology of transferring the pattern from the mask to the substrate by illuminating a photoresist film with a light source, *e.g.*, ultraviolet light.^4^ Photoresists are light sensitive materials that can undergo structure evolution after light irradiation. A typical photolithography process includes the following steps^5^ (Fig. 1): firstly, the photoresist solution is applied on the surface of a cleaned substrate (*e.g.*, ITO, silicon wafer) and forms a uniform thin film *via* spin coating, then the mask printed with the target pattern is placed between the light source and the photoresist layer to conduct the exposure. The light can pass through the transparent areas on the mask but not the opaque areas, so photo-induced conversion on the photoresist film will happen. After that, the solubility of the photoresist in areas where photoreaction occurs and areas where photoreaction does not occur would be different, and high soluble areas could be cleaned with a solution called developer to leave the pattern we need on the photoresist. If the photo-reactive areas solidify and the unexposed areas dissolve, this type of photoresist is called negative photoresist as shown in Fig. 1e, if areas where photoreaction occurs are more easily dissolved, unexposed areas would stay, and this type of photoresist is defined as positive photoresist as shown in Fig. 1f. Photoresists can serve as a protection layer to keep materials underneath intact during subsequent fabrication process. For example, it can avoid the etching of areas covered with the photoresist during the etching process (Fig. 1g). Finally, the target pattern is transferred to the substrate by a combination of lithography and etch process.

The overall process of lithography in principal is simple as discussed above, however, in practice, lithography quality is affected by many complicated parameters. Photoresists are one of the very important components in lithography. Photoresists can be classified as organic or inorganic according to their major composition. Conventional organic resin has been used as photoresist for semiconductor manufacturing. However, organic photoresists suffered from disadvantages such as low tolerance to etching conditions, especially dry etch conditions, low absorption efficiency to advanced lithography light source EUV. Inorganic material based photoresists have emerged as alternatives due to their high etch resistance and appropriate absorption to light sources. Ober and co-workers reviewed EUV resist materials for sub-7 nm patterning,^3^ and they also summarized their representative research work about metal oxide nanoparticle photoresist.^6^ It has been shown that metal oxide based photoresists are potential candidates for advanced photolithography, especially EUV lithography. The current review aims to focus on recent progress and opportunities about inorganic photoresist materials, including their fabrication process, performance and the working mechanism.

## 2. Factors that influence the photolithography

### 2.1 Factors that affect the resolution of photolithography process, *i.e.* the smallest size that could be manufactured in the photolithography technology process

The required size is different in different applications: in semiconductor manufacturing industry, we want to increase the number of transistors per unit area, so reducing the size of structures and improving the resolution are critical.^7^ However, membranes with micron injection units for medical use are not required to be too small. Theoretically, the resolution of Critical Dimension (CD) that photolithography could produce is related to Rayleigh formula: CD = *k* × *λ*/NA, *k* is a formula related to photolithography process, *λ* is the wavelength of the light source, NA is the numerical aperture of the lens.^8^ Therefore, reducing the wavelength of the light source could improve the resolution of photolithography technology and obtain a smaller structure. The wavelengths of several representative light sources used in lithography are shown in Table 1.

**Table 1:** Types of light sources required for photolithography technology process and corresponding wavelength range

Light sources	Categories	Wavelength nm	Spectral band range
Mercury arc lamp	g line	436	Visible light
Mercury arc lamp	h line	405	Visible light
Mercury arc lamp	i line	365	Medium ultraviolet
Excimer laser	KrF	248	Deep ultraviolet
Excimer laser	ArF	193	Deep ultraviolet
Excimer laser	Immersion ArF (water as medium)	132	Deep ultraviolet
Laser generated plasma	EUV	13.5	Extreme ultraviolet

### 2.2 Selection of light source and photoresist

**Fig. 2:**
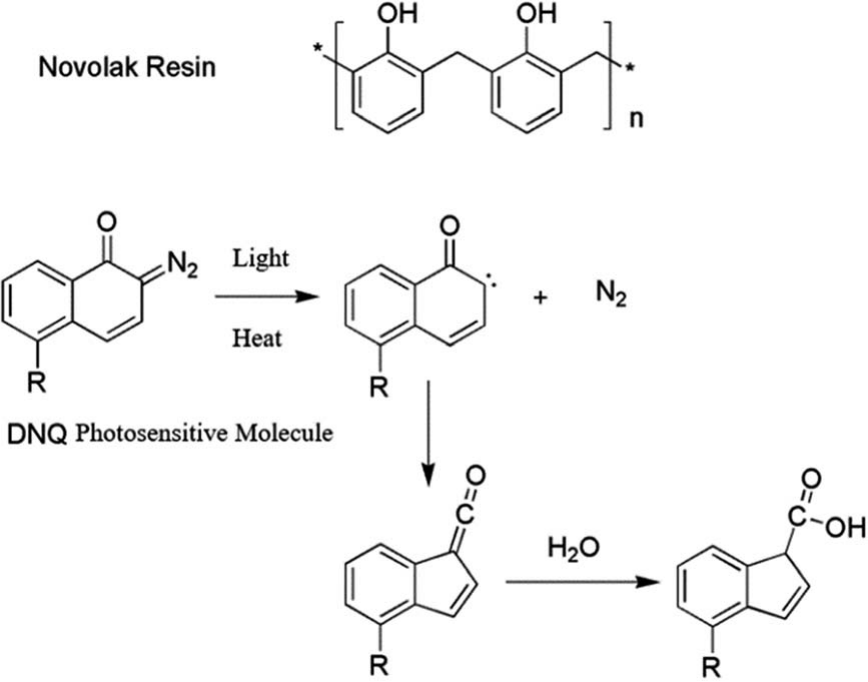
Basic resin structure of g-line and i-line photoresist and related photoreaction of photosensitizer.

**Fig. 3:**
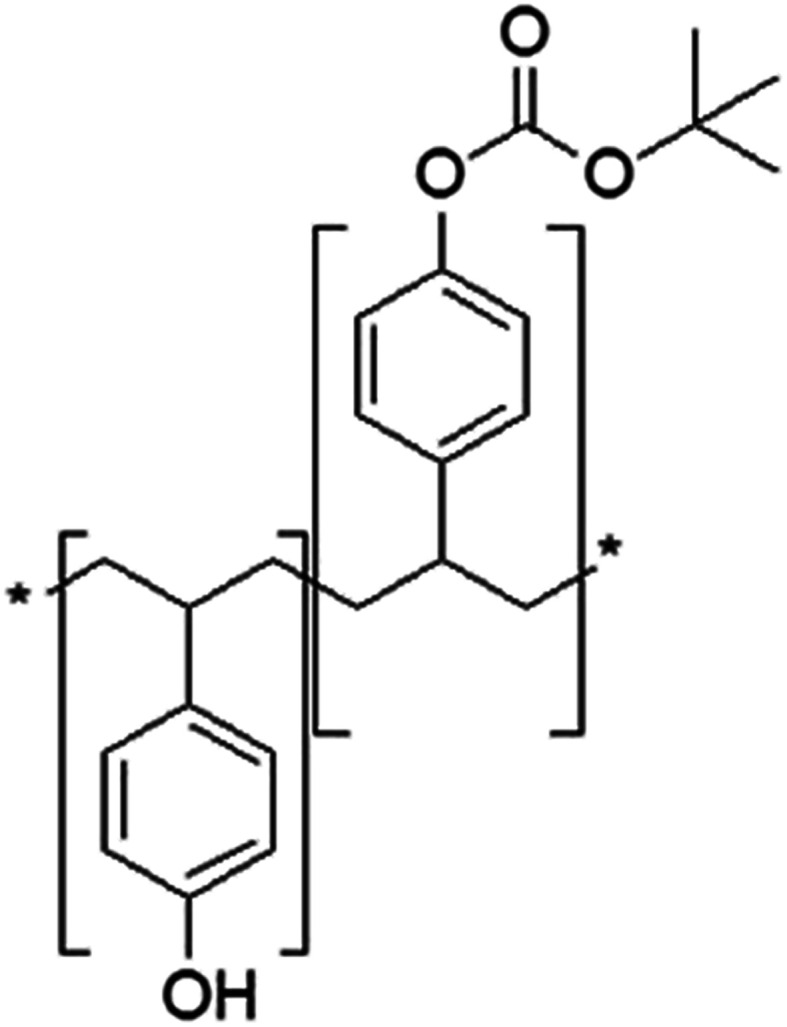
Basic structure of DUV photoresist resin with acid unstable groups.

In order to get efficient photolithography performance, the characteristics of light source used must be considered when looking for suitable photoresist. The g-line and i-line (i-line refers to 365 nm, g-line refers to 436 nm, and they are the two spectral lines with the highest energy in high-pressure mercury lamp) photoresists are mainly composed of novolak resin (phenolic resin), photosensitizer and solvent. The solubility of photosensitizer molecule diazonaphthoquinone (DNQ) in developing solution is small, so the existence of DNQ molecule in resin decreases the overall solubility. After light exposure, the release of nitrogen generates carbene structure to undergo intramolecular rearrangement, and active ketene structure is obtained, which easily reacts with water to form molecule easily soluble in dilute alkali solution.^9^ The basic structure of resin and photoreaction of sensitizer are shown in Fig. 2. This kind of photoresist is not suitable in DUV band, because novolak structure absorbs too much light, and the bottom part of the photoresist film could not absorb enough photons for photoreaction, thus the structure cannot be further developed with developer. Moreover, the light source power of DUV is lower compared with g-line and i-line. If the photoresist sensitivity is not enough, the exposure time needs to be extended, resulting in prolonged production cycle. To overcome the above problems, poly(*p*-hydroxystyrene) is developed for DUV lithography application (see Fig. 3). Their polymer backbones could be modified to improve the performance of photoresist, for example, modification based on acid catalyzed chemical amplification principle solves the problem of low sensitivity of photoresist. In this kind of photoresist, some photo-induced acid-generator additives (PAG) would produce a small number of acid after exposure. The acid could catalyze the decomposition of acid unstable groups in photoresist during the subsequent heating process, and the resulting polymer can be dissolved by the developer more easily. As a catalyst component, only a small amount of acid could amplify the exposure effect through chemical reaction, thus the sensitivity of photoresist greatly improves. This strategy was invented at IBM.^10^ However, if such catalysts are exposed to base contamination, such as organic amine compounds in the air, the catalytic process would be deactivated. Or if the underlying substrate is inorganic nitride, a small amount of amine substances might release during the preparation process, resulting in failure of photoresist development. There are several strategies to avoid alkali interference by controlling the source of amine contamination: various gases used are filtered, DUV exposure and development equipment is made by special materials that could not release trace amine substances during the manufacture process.

In order to print smaller patterns in semiconductor manufacturing process, the resolution of photolithography must be improved. EUV light source has a wavelength of only 13.5 nm. According to Rayleigh formula, the resolution of photolithography could be greatly improved. In comparison with conventional lithography, the EUV photoresist requires different properties.

### 2.3 The dose required for exposure

The maximum output power of the light source is constant, and the exposure dose could be adjusted according to the length of exposure time. Very sensitive photoresists require only a small dose to produce sufficient photoreaction, resulting in significant differences in dissolution between exposed and unexposed areas. Therefore the exposure time could be reduced, which is beneficial for improving production efficiency. Because EUV light source has lower wavelength and smaller power output, the development of highly sensitive photoresist is getting more and more important.

### 2.4 Development

**Fig. 4:**
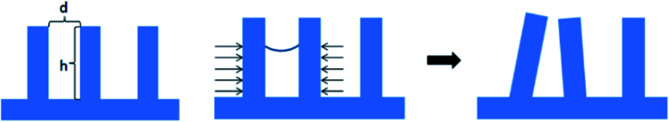
The schematic view of the aspect ratio of a photoresist pattern and a schematic view of the pattern collapse due to the surface tension of developer.

For the development process after exposure, dilute alkaline solution, such as tetramethylammonium hydroxide, is generally used, and potassium hydroxide or sodium hydroxide is avoided, because these metal ions would affect the electrical properties of the transistor. After development, a corresponding three-dimensional structure could be obtained on the photoresist, and *h*/*d* is represented as the aspect ratio of the structure, where *h* is the height and *d* is the space dimension of the pattern (see Fig. 4). The next step of photolithography process could be etch or implantation, so photoresist needs to tolerate the reaction conditions of substrate etching or ion implantation. Organic-based photoresists tend to have low density and need to have enough thickness to effectively protect the underlying substrate from etching and ion implantation. However, when the aspect ratio is too large, the strength of organic photoresists would be insufficient, and the surface tension of developer would easily lead to pattern collapse (Fig. 4). To overcome this problem, the main solution is to add appropriate surfactant into the developer or further solidify the photoresist by heating to avoid collapse. In view of the above limitations of organic photoresists, inorganic-based material would have more advantages. First of all, inorganic materials are more resistant to etching, especially dry etching like plasma etching. Only a thinner layer of photoresist is sufficient to protect the underlying material from etching and ion implantation. Secondly, because inorganic materials are structurally more stable than organic polymers, even if the aspect ratio is slightly larger, it is not easy to cause pattern collapse.

### 2.5 Line edge roughness (LER) of photoresist

LER refers to the roughness of photoresist edge after lithography. Optimized process and photoresist materials are required to ensure that the prepared structure edge is flat and smooth. The LER after photoresist development would further affect the variation degree of CD generated by etching the underlying substrate. The smaller of the pattern we want to make, the higher requirement for LER.

### 2.6 Overall properties of photoresist

The comprehensive performance of photoresist could be evaluated by RLS: namely, R (resolution), L (LER or LWR), S (sensitivity). All these factors are crucial. To meet the requirements of industrial production, photoresist need to meet the following metrics: resolution < 10 nm, LWR < 15%, sensitivity < 20 mJ cm^−2^.^11,12^

## 3. Inorganic photoresists

To continuously increase the performance and reduce the power consumption simultaneously for portable electronic products, the development of more advanced photolithography technique is significant. On the basis of the above discussion, EUV was the key technology to push the semiconductor manufacturing process to a smaller size, and the development and research of photoresist materials is an essential part of EUV technology. Due to the low energy of EUV light source, photoresist with better sensitivity was needed. According to the principle of DUV chemical amplified photoresist, researchers developed photoresist based on organic substances. Fujii's team from Japan evaluated the factors affecting the performance of chemically amplified photoresist.^13^ The acid generation efficiency can be improved through increasing the proton source content in the polymer, or involving stronger electron-withdrawing substitution group on PAG cation due to higher quantum yield. By changing the formulation of photoresist, adjusting the amount of *T*_g_ enhancer unit (used to inhibit acid diffusion pathway), the proton source unit in the polymer, and the electron withdrawing group in PAG, lithographic performance could be tuned. With several optimal formulations, 13 nm hp LS (half pitch line/space) pattern was obtained on NXE3300 EUV scanner, 12 nm hp LS was obtained on interference lithography tool at PSI, and ×16 nm contact hole was obtained on NEX3300. Considering the RLS trade-off, these resist formulation optimizations were helpful to the HVM (high volume manufacturing) of EUV lithography. Hori *et al.* found that controlling the acid diffusion distance of PAG could improve the RLS of photoresist in EUV lithography.^14^ Addition of photosensitizer with better EUV absorption into photoresist was also helpful to improve EUV utilization, promote secondary electron generation more effectively, and then trigger PAG activation with the help of secondary electrons. Therefore the sensitivity of photoresist was improved.

Traditional chemically amplified organic photoresists had some limitations. Firstly, when the size of the structure to be prepared was very small, the film thickness of EUV photoresist must be small (∼35 nm) in order to avoid pattern collapse caused by high aspect ratio. At this thickness, organic photoresist was difficult to tolerate etching conditions. Moreover, the absorption coefficient of 35 nm thick organic photoresist to EUV was 4.8 μm^−1^, a value corresponding to the utilization of 15% EUV energy, resulting in significant waste for expensive EUV irradiation. Secondly, the RLS trade off for organic photoresist in EUV lithography technology was also unsatisfactory. On the other hand, inorganic photoresist materials with different EUV absorption coefficients and high etch-resistance are leading candidates to solve some existing problems. Therefore, more and more researchers have begun to study the application of inorganic materials in photoresist field.

**Fig. 5:**
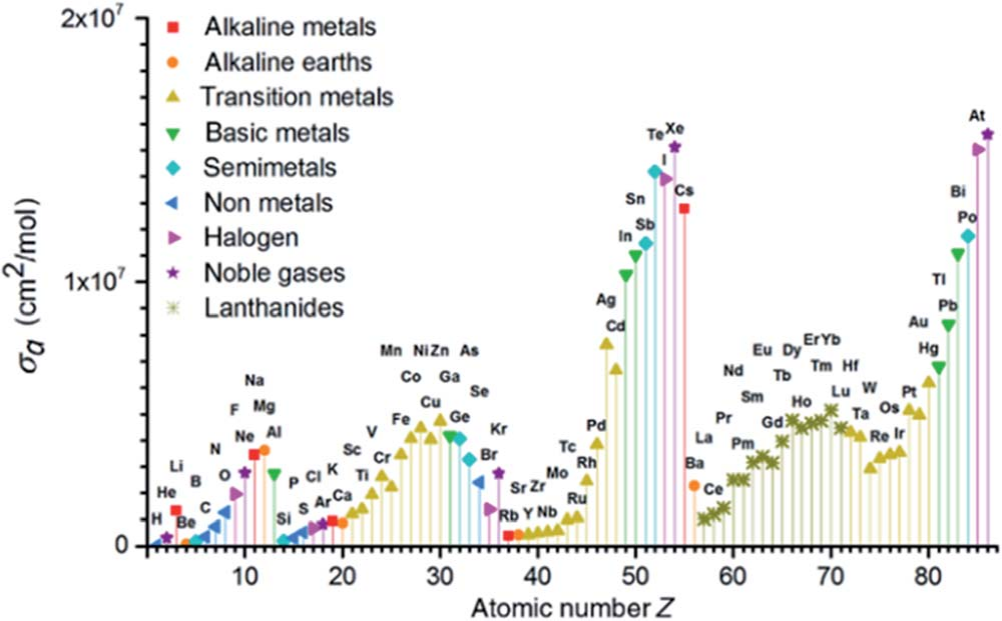
Atomic absorption cross section *σ*_a_ of EUV light source with atomic number *Z* from 1 to 86. Reprinted with permission from ref. 15. Copyright 2018 SPIE.

In photolithography technology, the photoresist film must have appropriate absorption to the light. Most importantly, it must have sufficient light transmittance to ensure that the entire film could be exposed from top to bottom. In addition, the film must effectively absorb and utilize the energy of incident light. Compared with DUV, EUV photon energy was higher, and less than one tenth of EUV photon is equivalent to the same exposure dose as DUV. Many different elements can absorb EUV light (see Fig. 5). Fallica's team reported the absorption coefficient of EUV light by different metal-containing photoresist films.^15^ They found that the absorption coefficient of tin clusters and organotin compounds was 2–3 times than that of traditional CAR organic photoresist, the absorptivity of ZrO_*x*_ was basically the same as that of organic photoresist, and the absorptivity of HfO_*x*_ was about twice as compared with ZrO_*x*_. This was related to the larger atomic absorption cross section of Hf to EUV. These measurement results had laid a theoretical foundation for the research of EUV photoresist. In DUV photolithography, after a molecule absorbed 193 nm photons, the electron absorbed energy in the same molecule and transited from the ground state to the excited state. However, in EUV photolithography, after the molecules absorbed high energy (92 eV) EUV photons, the electrons in the molecules would separate from the molecules with 75–82 eV energy, leaving holes of positive ion radicals in the molecules. These electrons had sufficient energy to further undergo ionization reaction or other reactions and continue to generate secondary electrons (2–80 eV). Chemical reactions caused by electrons and holes could change the solubility of photoresist. Studying the reactions of electrons and holes with photoresist was very important for the development of EUV photoresist.^16^

### 3.1 IVB group (Hf, Zr, Ti) photoresists

Ober *et al.* published an article in 2010 about the use of HfO_2_-based inorganic photoresist for DUV, EUV and E-beam lithography.^17^ Hafnium isopropoxide was used as reactant, after hydrolysis and condensation reaction, the obtained white precipitate was washed with acetone and dried, finally the materials were dissolved in propylene glycol monomethyl ether acetate (PGMEA) solvent to prepare photoresist solution. The ligand on the surface could be replaced by other carboxylic acid through ligand exchange reaction, and the PGMEA solution need to be filtered through a 200 nm membrane to remove large particles. The particle diameter of HfO_2_ in the solution was between 1–3 nm, such small particle won't induce obvious light scattering effect, and the narrow particle size distribution was also conducive to the improvement of photolithography resolution. When 2,2-dimethoxy-2-phenyl acetophenone (DPAP) was used as photoinitiator for DUV lithography, it was first prebaked at 130 °C for 60 seconds, and then DUV lithography (254 or 193 nm) was performed. The development in IPA showed the property of negative photoresist. If post exposure bake (PEB) was added at 130 °C for 3 minutes after exposure and then developed with TMAH alkaline aqueous solution, it could be used as positive photoresist. Compared with PHOST (poly-hydroxyl styrene) film, the etch resistance of photoresist containing hafnium oxide was improved by 2–3 times, and the etch resistance could be further enhanced by reducing the content of organic contents in the photoresist. This kind of photoresist could also be used for E-beam lithography. In 2011, Ober *et al.* published a HfO_2_ photoresist with higher etch resistance,^18^ the detailed formulation had not been reported, which might be related to organic ligands that could undergo cross-linking reaction upon the irradiation of light. The etch resistance was 9 times than that of PHOST. The etch resistance could be further increased to 43 times than that of PHOST by adding one step of baking after development. If O_2_ plasma treatment was performed after development, the etch resistance could be increased to 68 times than that of PHOST. Similar to hafnium oxide, zirconium oxide could also be used as inorganic photoresist. Ober *et al.* also reported ZrMAA photoresist.^19^ For DUV, E-beam, EUV lithography, this photoresist could realize the function of positive- and negative-tone imaging by changing the process conditions.

**Fig. 6:**
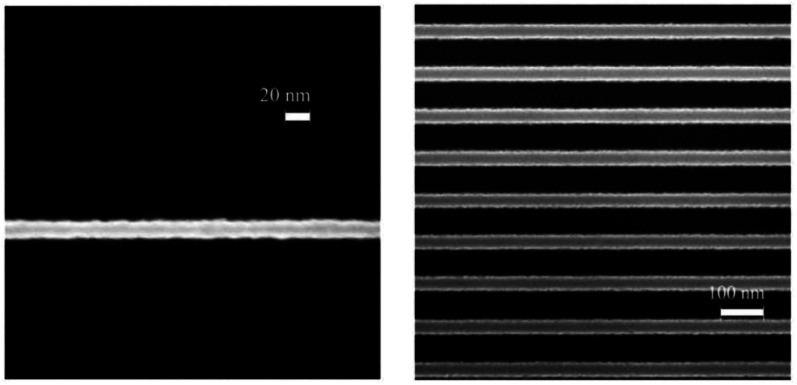
Left: SEM image of a 15 nm isolate line in ZircSO_*x*_ at a dose of 999 μC cm^−2^, right: SEM image of 36 nm lines on a 100 nm period in ZircSO_*x*_ at a dose of 810 μC cm^−2^ (30 keV electron-beam). Reproduced with permission from ref. 20 Copyright 2009, Elsevier B. V.

Stowers' research team reported ZircSO_*x*_, HafSO_*x*_ photoresist with high sensitivity for E-beam lithography.^20^ As shown in Fig. 6, at 30 keV, the photoresist sensitivity reached 8 μC cm^−2^, and could print 15 nm isolated line (999 μC cm^−2^) and 36 nm dense features (810 μC cm^−2^). In plasma etching, the etch resistance of the photoresist was 7 times than that of silicon dioxide. The preparation process of the photoresist comprised the following steps: solution containing ZrOCl_2_·8H_2_O, HfOCl_2_·8H_2_O, H_2_SO_4_, H_2_O_2_ and ultrapure water was prepared. And the metal-peroxide ratio was 1 : 0.5, metal-sulfate ratio was 1 : 0.5–0.7, metal concentration was 0.5 M, the thickness of spin coated film was 35 nm. An SEM equipped with Nabity lithography system was used for the exposure process, and 25% TMAH was employed as the developer. The final baking temperature was 325 °C for 5 minutes. The mechanism of the negative tone imaging was due to the presence of hydrogen peroxide in the system. The existence of hydrogen peroxide could inhibit the aggregation reaction of metal oxide solution.^21^ Under the irradiation of electron beam, hydrogen peroxide decomposition can trigger the aggregation and cross-linking reaction of metal oxide compounds, which further lead to the difference in solubility between exposure and non-exposure areas.

**Fig. 7:**
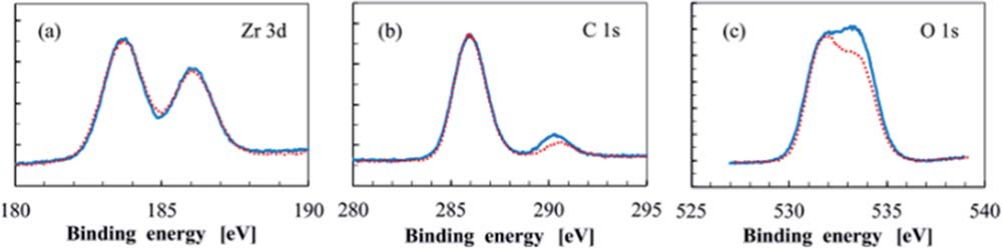
High resolution XPS spectrum of (a) Zr 3d, (b) C 1s, (c) O 1s in ZrO*_x_*–MAA metal photoresist, blue represents spectrum before EUV exposure, and red represents XPS after EUV exposure of 20 mJ cm^−2^. Reproduced with permission from ref. 22 Copyright 2016 SPIE.

Toriumi's team studied photoresist composed of metal oxides and organic molecules,^22^ including the composites of zirconium oxide (ZrO_*x*_), titanium oxide (TiO_*x*_) and methacrylic acid (MAA) ligands, the morphology of particles was characterized by STEM, and the surface composition of the materials was tested by XPS before and after EUV exposure (Fig. 7). STEM test found that ZrO_*x*_ core in ZrO_*x*_–MAA had better dispersibility, but TiO_*x*_ core in TiO_*x*_–MAA were more easily to agglomerate, which might be due to stronger interaction between ZrO_*x*_ core and shell molecules, in contrast, MAA and TiO_*x*_ had stronger interaction with themselves. XPS confirmed that ZrO_*x*_–MAA photoresist had some surface ligand molecules decomposed after exposure, and the electron density of Zr atoms increased after EUV treatment. The Ober's team also used FTIR and XPS to confirm that Zr–MAA would lose some carboxyl ligands after EUV exposure in their study.^23^

**Fig. 8:**
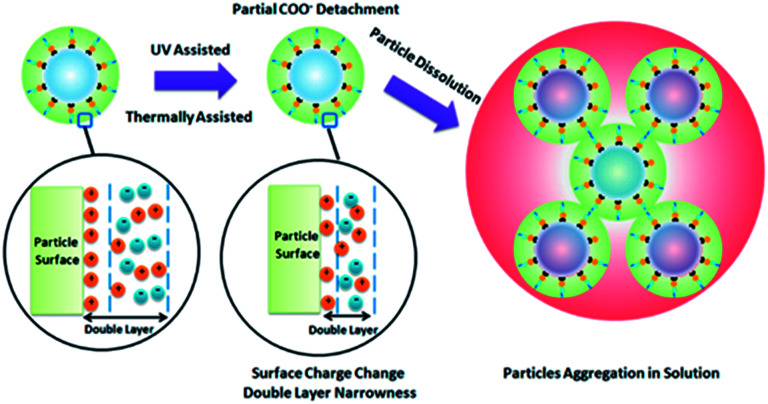
The mechanism of HfO_2_ particle size increase after UV treatment. Reproduced with permission from ref. 24. Copyright 2015, American Chemical Society.

Li *et al.* studied the light conversion mechanism of nano-particle HfO_2_ hybrid photoresist with different surface ligands (MAA methacrylic acid, DMA dimethacrylic acid, BA benzoic acid) under DUV 254 nm ultraviolet light.^24^ By comparing the properties of the particles before and after illumination with TGA, XPS and DLS, it was concluded that UV illumination caused a small amount of ligand dissociation from the surface of the metal particles, which can result in the change of surface charge on the metal oxide nanoparticles, and further lead to aggregation, increase of particle size and change of solubility of the metal nanoparticles (Fig. 8). MAA and DMA ligand modified HfO_2_ were easier to agglomerate under UV than BA modified HfO_2_. The authors believed that this was due to the stronger interaction between BA and metal oxide particles, as a consequence, the metal oxide particles/ligands formed were more stable.

**Fig. 9:**
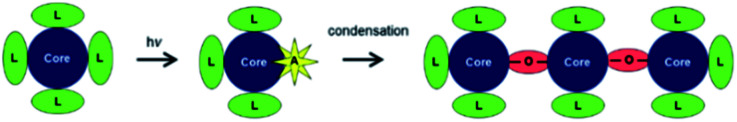
Basic model of metal oxide photoresist imaging. Reproduced with permission from ref. 25. Copyright 2017 SPIE.

Hinsberg *et al.* established an imaging model of metal oxide photoresist by theoretical simulation method.^25^ The model provided a complete simulation of the chemical reaction of this kind of photoresist during exposure and subsequent thermal treatment. Due to the activation of light irradiation, the metal oxide core lost some ligands, aggregated to form insoluble oxide aggregates with some M–O–M chemical bonds (Fig. 9). This mechanism is consistent with the negative tone imaging observed in experiment. The model they created could predict the lithography contrast, LWR, exposure dose and other results that the photoresist could achieve with some input parameters. The model was also verified by the experimental results of a photoresist formulation developed by Inpria company and the model suggest that nonlinear growth of an oxo-network when exposure dose increases is the origin of negative-tone lithographic contrast.

Although some reports believed that some ligands on the surface of inorganic nanoparticles dissociated after exposure, resulting in aggregation of inorganic nanoparticles. Some studies also had different views. Castellanos *et al.* studied the properties of Ti, Zr, Hf Oxygen clusters [Ti_8_O_8_Mc_16_, Zr_6_O_4_ (OH)_4_Mc_12_, Hf_6_O_4_ (OH) _4_Mc_12_ (HOBu), Mc = methacrylate, Bu = butyl] with exact molecular weight before and after EUV exposure.^11^ They were abbreviated as TiMc, ZrMc, HfMc respectively. When the powdered samples were deposited as thin films, ZrMc and HfMc lost some carboxylic acid ligands that are not bonded with metals by covalent bonds. Thermogravimetry proved that in the crystalline powder sample, the actual chemical formulas were ZrMc–3McOH and HfMc–2.5Mc respectively, while the TiMc sample did not lose carboxylic ligands when preparing thin films. In powder crystals, the ligands that were not covalently bonded with metals participate in stabilizing clusters. After forming a film, the absence of these ligands caused the clusters more sensitive to hydrolysis or rearrangements under certain conditions (*e.g.*, exposure). In EUV lithography experiments (using chloroform as developer), researchers found that the sensitivity of HfMc was higher than that of ZrMc, and this trend was consistent with the absorption coefficient of them, *μ*(HfMc) = 9 μm^−1^, *μ*(ZrMc) = 5 μm^−1^. However, the unexposed TiMc film couldn't be developed by chloroform or general organic solvent.

The author further studied the mechanism of photo-resist solubility change caused by exposure and found a small amount of ligand loss through XPS measurement. For ZrMc, there were 12 ligands per cluster. After exposure at a dose of 50 mJ cm^−2^, at most 1.4 ligands were lost, while HfMc detected 19% ligand loss after exposure at a dose of 250 mJ cm^−2^. These data showed that when the solubility of photoresist changed, the loss of ligands on the surface was not obvious. At the same time, Grazing Incident X-ray Scattering (GIXS) was used to measure photoresist film, it was found that the particles were arranged in a disordered state, and the aggregation of particles was insignificant, indicating that this kind of metal oxide photoresist did not require significant ligand loss or extreme polymerization to realize solubility change after exposure. Changes in solubility might be related to other factors, such as coupling of terminal carbon–carbon double bonds.

### 3.2 Tin–oxygen cluster inorganic photoresists

**Fig. 10:**
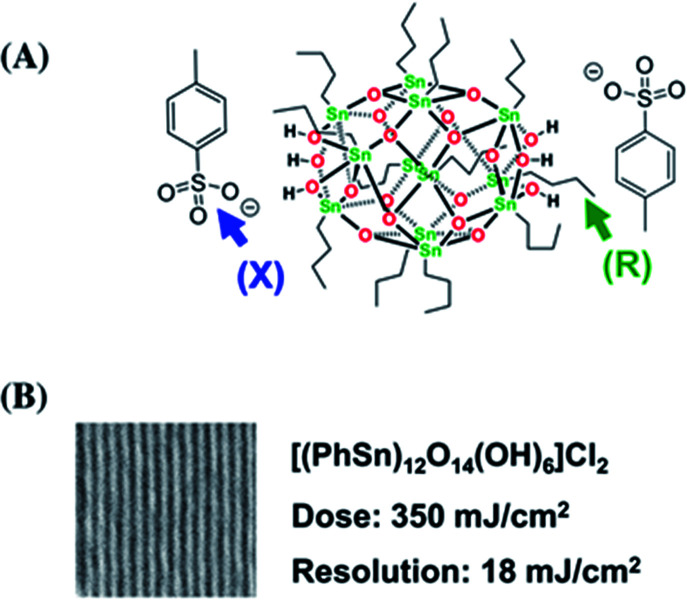
(A) [(BuSn)_12_O_14_(OH)_6_]X_2_ structure, the performance of metal clusters in EUVL was screened by changing anion X and organic ligand R attached to Sn. (B) Lithography results of the best metal cluster obtained by screening, wherein the organic substituent on Sn was benzene, and the anion was chloride ion. Reproduced with permission from ref. 26 Copyright 2014, Elsevier B. V.

**Fig. 11:**
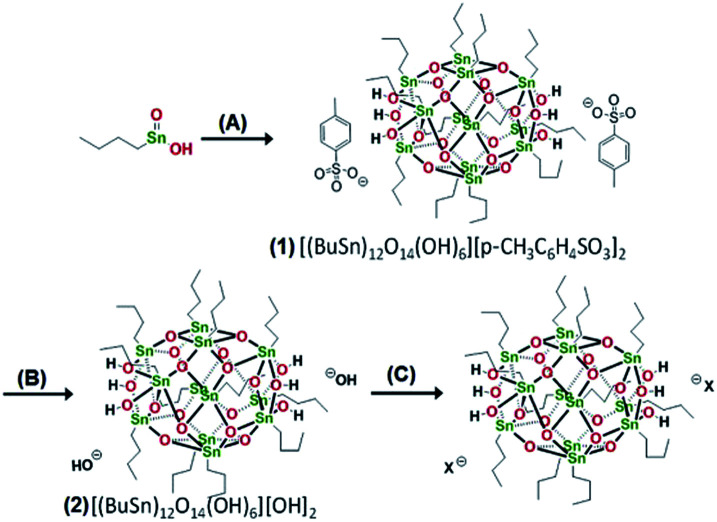
Anion exchange reaction to change carboxylic acid X anion in cluster. Reproduced with permission from ref. 26. Copyright 2014, Elsevier B. V.

Brainard's team reported for the first time the lithographic performance of [(BuSn)_12_O_14_(OH)_6_]X_2_ metal clusters under EUV.^26^ The absorption density of EUV photons by Sn and O elements was 10.5 times and 1.7 times higher than carbon, so these two elements could make better use of EUV photons in EUV lithography technology. Tin-oxo clusters were smaller and more uniform than HfO_2_ nanoparticles, thus theoretically they had better resolution and LER. EUV irradiation can activated the photoreaction of organic ligands on the surface of tin clusters reported in this study, resulting in the aggregation of clusters and then showing the performance of negative photoresist. The cluster structure was shown in Fig. 10. The author changed the type of carboxylate anion through anion exchange reaction (Fig. 11). It was found that the bond strength between α-carbon and carboxyl in carboxylic acid had no correlation with the sensitivity of photoresist, but the higher the relative molecular mass of carboxylate anion, the worse the sensitivity of photoresist. Therefore, it was inferred that the photoreaction might occur on tin-oxo clusters. Although anions did not participate in the photoreaction, they could hinder the aggregation of clusters due to steric hindrance. Another mechanism of photoreaction was related with the dissociation of Sn–C bond to form free radicals, since radicals formed by tin were relatively stable, the stability of organic radicals generated by photodecomposition would affect the photosensitivity of tin-oxo clusters. The results showed that the more stable the radicals formed by the organic structure substituted on tin were, the higher the sensitivity of photoresist was. It was concluded that the photoreaction mechanism of tin-oxo was particle agglomeration caused by dissociation of ligand on tin. Although the sensitivity of this tin cluster compound was poor, the author believed that high-resolution lithography resists could be prepared by using ligands with higher sensitivity or other material systems.

In EUV and DUV and E-beam lithography, tin-oxo could be used as negative photoresist. For EUV, tin-oxo was expected to obtain a high-resolution structure due to its high absorption, small particle size and high sensitivity. The process conditions of tin-oxo cluster as negative photoresist were: EUV dose of 30 mJ cm^−2^, PEB (100 °C for 2 min), and development (2 : 1 IPA/H_2_O for 30 s).^26^ Zhang's team found that by changing the process conditions,^27^ [(BuSn)_12_O_14_(OH)_6_](OH)_2_ could be used not only as positive photoresist but also as negative photoresist. The process conditions were: PEB (150 °C for 2 min), development (2 : 1 IPA/H_2_O for 30 s), positive tone imaging was obtained at low exposure dose and negative tone imaging was obtained at high exposure dose. At a high dose (250 mJ cm^−2^), a large amount of organic ligands were decomposed (confirmed by XPS characterization), which lead to the aggregation of tin-oxo particles and showed the property of negative photoresist. However, at a low dose (*e.g.* 10 mJ cm^−2^), the author thought that EUV or electron beam might make the particles insensitive in the subsequent PEB (PEB could reduce the solubility of tin-oxo), and the solubility decrease in the exposed area was not obvious enough. Therefore, the positive photoresist performance was reflected in the same developer, and the exact mechanism was still under study.

**Fig. 12:**
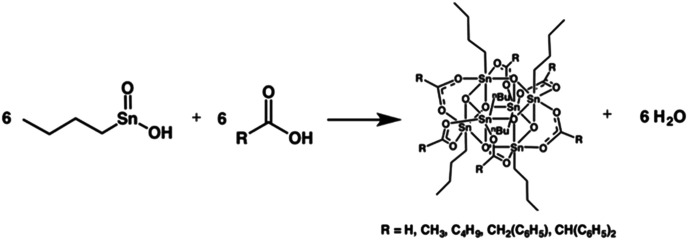
Organotin compound [RSn(O)O_2_CR′]_6_. Reproduced with permission from ref. 28. Copyright 2017, Wiley-VCH Verlag GmbH & Co. KGaA.

Johnson's team studied the crystal structure of a series of organotin compounds [RSn(O)O_2_CR′]_6_ by single crystal X-ray diffraction.^28^ The structure was shown in Fig. 12. Tin has good absorption for EUV and excellent selectivity for etching. When such materials come into contact with EUV light sources, the organic ligands on the tin surface would decompose, causing the tin clusters to undergo crosslinking reaction. Therefore, the ligand on the surface played a very important role, which not only affected the interaction and stacking structure of clusters in the film structure, but also could regulate the electronic structure of tin metal and change its sensitivity to EUV light source. This kind of photoresist belonged to negative-tone photoresist.

Inpria also developed tin-based photoresist.^29^ The formulation of photoresist was adjusted to optimized the absorption of EUV photons and ensure that resist had sufficient light transmittance to allow EUV to interact with the bottom part of photoresist while maximizing EUV photon absorption. The optimal formulation could realize the lithographic performance of 13 nm HP at a dose of 35 mJ cm^−2^. The detailed formulation of the photoresist was not documented in the paper.

### 3.3 Zinc-based inorganic photoresist

**Fig. 13:**
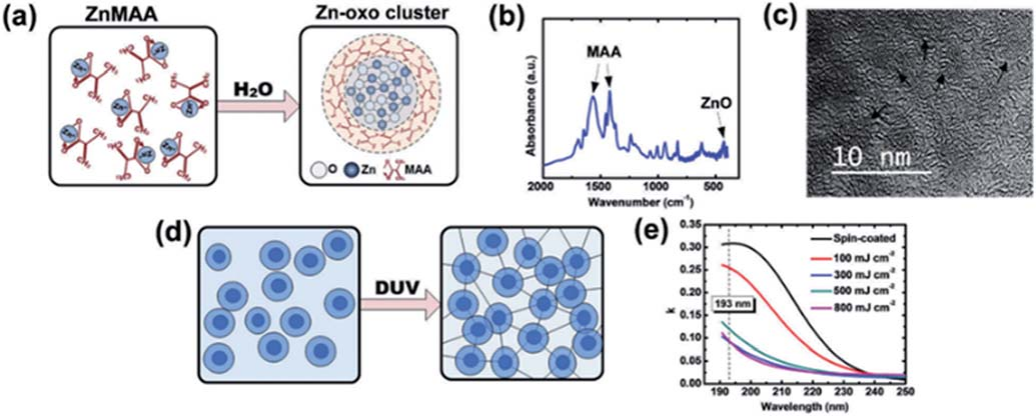
(a) Formation of zinc oxide clusters (ZnOCs) by hydrolysis–dehydration reaction. (b) FTIR of ZnOC films. (c) HRTEM pictures of ZnOCs particles in photoresist. (d) Schematic diagram of DUV excited ZnOCs crosslinking. (e) Absorption curves of ZnOC films under different DUV light doses. Reproduced from ref. 30 with the permission of the Royal Society of Chemistry.

ZnO can also be used as metal oxide photoresist.^30^ Zinc oxo cluster ZnOCs was synthesized by hydrolysis–condensation of zinc methacrylate (ZnMAA) precursor. After DUV exposure, the ligand of zinc oxo clusters was detached from the surface to induce the aggregation of inorganic cores in the exposed area, which lead to the decrease of solubility in organic developer and showed negative-tone imaging property. The synthetic method and photoreaction mechanism of ZnOCs were shown in Fig. 13.

**Fig. 14:**
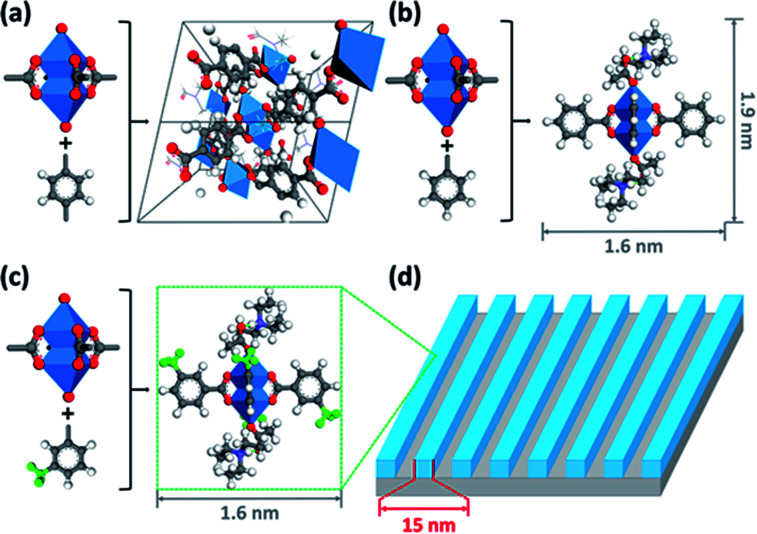
MOF-2, Zn-BA and Zn-mTA clusters constructed using Zn_2_(CO_2_)_4_ containing metal units and organic ligands. (a) Crystal structure of MOF-2. (b) Structure of Zn-BA. (c) Structure of Zn-mTA. (d) A 15 nm HP LS structure could be obtained by photoetching technology using Zn-mTA as photoresist. Atomic color: C – gray. O – red. N – purple. H – white. Zn – blue. Reproduced with permission from ref. 31. Copyright 2015, American Chemical Society.

Inspired by the structure of MOF-2, Ober's team designed and synthesized metal clusters containing zinc for EUV lithography (Fig. 14).^31^ A photoresist that could be used for EUV lithography was obtained (Fig. 14). MOF-2 was a microporous material connected by Zn_2_(CO_2_)_4_ MBU (metal containing building units) through four styrene structures. Ober's team synthesized phenyl modified Zn_2_(CO_2_)_4_ and 3-methylphenyl modified Zn_2_(CO_2_)_4_, which were respectively called Zn-BA and Zn-mTA, wherein Zn-BA could obtain single crystal structure. However, crystallization was an unfavorable factor in the preparation of photoresist films, which would bring difficulties to high-resolution lithography. The Zn-mTA thin film could obtain a 1 : 1 LS structure of 16, 15, 14, 13 nm at EUV doses of 45, 47, 36, 35 mJ cm^−2^. The edge roughness at high resolution (15 nm) was obviously better than that of zirconia and hafnium oxide nanoparticles, which might be due to the smaller particle size and more uniform structure of Zn-mTA clusters. The Zn-mTA photoresist also contained PAG and low boiling point ligands acetic acid and triethylamine. The acid generated by PAG could be used as ligand exchanger on the surface of metal clusters to trigger the solubility change.

**Fig. 15:**
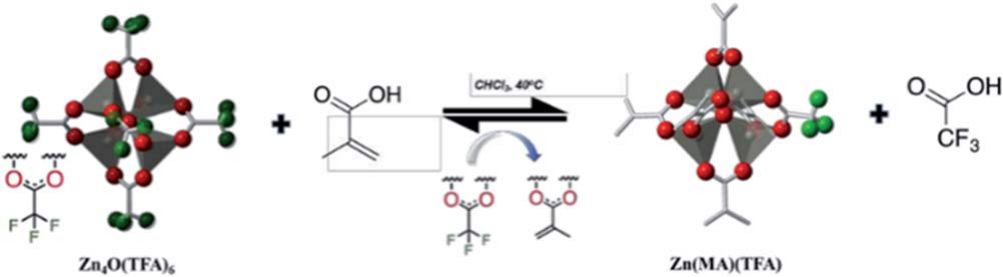
Synthesis of Zn(MA)(TFA) clusters by using methacrylic acid (MA) to replace trifluoroacetate (TFA) on the surface of Zn_4_O(TFA)_6_. Reproduced with permission from ref. 12. Copyright 2019 SPIE.

Thakur *et al.* studied the application of zinc containing clusters as photoresist in EUV lithography.^12^ Zinc had high EUV photon absorption cross section. As shown in Fig. 15, by changing the surface ligands of zinc containing oxocluster to different molecules, the solubility and photo reactivities of nanoparticles were tuned. The addition of fluorine atoms improved the EUV absorption of the system, while the double bond in methacrylic acid could significantly affect the solubility of cluster due to polymerization. The synthesis of Zn(MA)(TFA) was realized by ligand exchange between Zn_4_O(TFA)_6_ and methacrylic acid. The structure and composition of the synthesized oxoclusters were confirmed by mass spectrometry, elemental analysis and thermogravimetry. Zn(MA)(TFA) oxoclusters would undergo dissociation of a small amount of acid ligands within 2 months, but the zinc core remain intact. After Zn(MA)(TFA) was deposited as a thin film, water vapor in the environment or in the thin film would hydrolyze part of Zn(MA)(TFA), and natural light in the environment would also cause polymerization of double bonds in MA, but placing the thin film in a vacuum environment (EUV exposure needed to be carried out in vacuum to avoid absorption of EUV by air) would not cause ligand dissociation. The photoresist could be used for EUV lithography to obtain a 30 nm HP LS structure, but different batches of Zn(MA)(TFA) required different EUV doses, which might be due to the weak bonding force of surface carboxylate ligand and zinc core, and lead to structural differences in each batch, although their compositions were consistent.

### 3.4 Inorganic photoresist containing aluminium

Nam's team prepared organic–inorganic hybrid positive photoresist containing aluminium.^32^ After spin-coating PMMA (polymethyl methacrylate) film on the substrate, trimethyl aluminum (TMA) was injected into the film by atomic layer deposition, and then steam was introduced to convert TMA into aluminum Oxide (AlO_*x*_). Through different numbers of cycles, the amount of AlO_*x*_ formed in PMMA could be adjusted. The composite photoresist had better contrast and etch resistance when applied to E-beam lithography. The contrast ratio of hybrid photoresist was 6 times that of standard PMMA, and the etch resistance selectivity to silicon was 70 times.

**Fig. 16:**
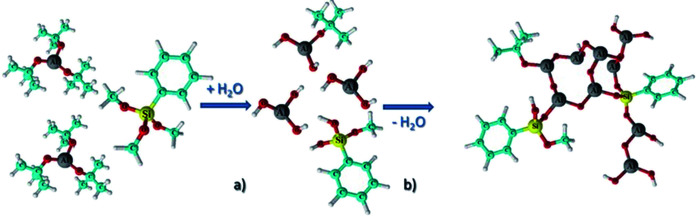
A metal organic precursor formed a partially aggregated alumina structure (the structure shown was a photoresist film before exposure) through hydrolysis and further dehydration polymerization. Reprint with permission from ref. 35. Copyright 2013, Wiley-VCH Verlag GmbH &Co. KGaA.

**Fig. 17:**
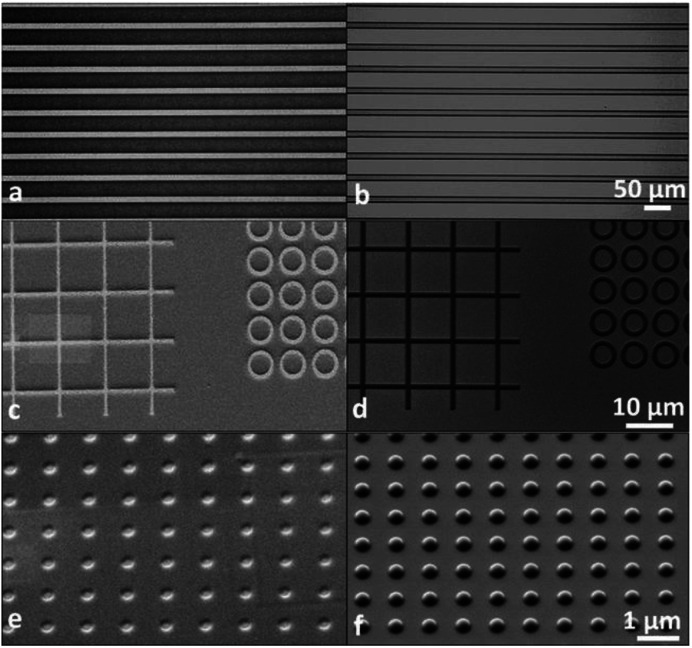
Optical and SEM images of the positive (a, c and e) and negative (b, d and f) developments obtained by UV (a and b), X-rays (c and d) and EB (e and f) lithography. Reprint with permission from ref. 35. Copyright 2013, Wiley-VCH Verlag GmbH &Co. KGaA.

Grenci and Brusatin's team also investigated the photoresist containing aluminium, and they found that addition of boehmite nanoparticles (aluminium hydroxide, γ-AlO(OH)) in a radiation sensitive sol–gel silica based system can lead to positive photoresist with high dry etch resistance (>60 when used for silicon etching).^33^ However, such a system is still not ideal due to some limitations pointed out in their following work,^34^ for example, the resolution is limited by the size of the nanoparticles, and the requirement of highly energetic X-ray radiation for uniform exposure and pattern, moreover, the resist system contains high content of absorbing components not sensitive to radiation, and the residual boehmite nanoparticles can decrease the quality of final pattern. To improve the resist performance, an aluminium-containing precursor, instead of nanoparticle, was used to synthesize alumina-like ceramic resist film after soft X-ray exposure and mild thermal treatment. The obtained organic–inorganic hybrid photoresist had a selectivity of 60 : 1 in the presence of ICP plasma etching. The photoresist with a thickness of 30 nm can tolerate the etch condition to etch more than 3 μm structure in the underlayer substrate.^34^ The research team also used aluminum-tri-*sec*-butoxide and a phenyl-modified silane reagent as the thin film precursor of photoresist (see Fig. 16).^35^ The performance of the resist in UV lithography and E-beam lithography was investigated (Fig. 17). By changing the process conditions, the system could show the image tone of positive photoresist and negative photoresist. The photoresist's structure was analyzed by FTIR before and after exposure, the author believed that the decomposition of organic components in photoresist lead to the aggregation of inorganic components and changed the solubility. In the fluorine-containing plasma etching test, the etch resistance of the photoresist compared with the underlying silicon was 100 : 1.

### 3.5 Metal organic complex molecular photoresist

**Fig. 18:**
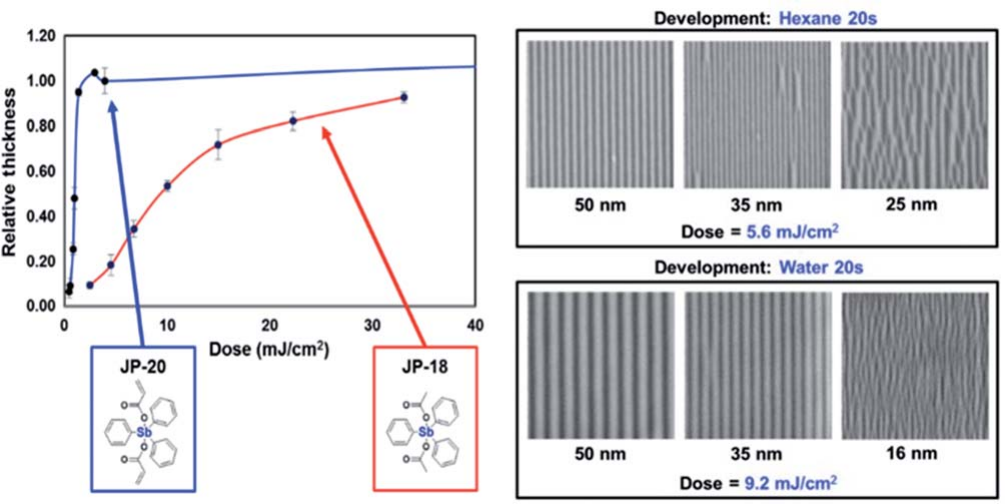
Left: comparing the sensitivity of metal organic molecular photoresists, the oxidation number of Sb metal in JP-20 and JP-18 was the same, and the substituents were also similar. The existence of double bonds played a great role in improving the sensitivity of photoresists. Right: lithographic performance of JP-20 is a representative molecule of this resist platform. High sensitivity at moderate resolutions is demonstrated. Ultimate resolution is limited by pattern collapse. Reprint with permission from ref. 36. Copyright 2015 SIPE.

**Fig. 19:**
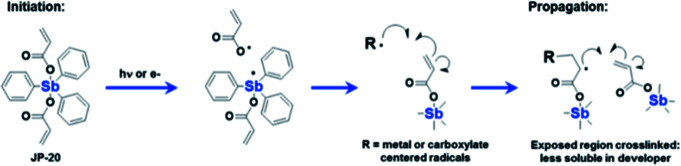
A possible mechanism of polymerization of Sb organometallic compounds under illumination. Reprint with permission from ref. 36. Copyright 2015 SIPE.

In addition to inorganic photoresists dominated by nanoparticles or nanoclusters, molecular organometallic compounds were also used in EUV lithography. Brainard's team developed a class of organometallic carboxylic acid compounds [R_*n*_M(O_2_CR′)_2_] that could be used as negative photoresist for EUV lithography.^36^ By changing the structure of R group, metal element (antimony, tin, bismuth) and carboxylic acid (such as acrylate, methacrylate, styrenecarboxylate) in the main molecules of photoresist, the author screened the performance of a series of compounds for EUV lithography. The most sensitive photoresist contained antimony, 3 R groups and 2 carboxylic acids containing polymerizable olefin double bonds (Fig. 18). It was believed that the high sensitivity was due to the double bond polymerization initiated in the exposed area (Fig. 19). A photochemical reaction could initiate a series of polymerization reactions and lead to changes in solubility, which might be the reason for its high sensitivity.

Other organometallic complexes, such as organotin, could also be used in EUV photolithography applications. Herman *et al.* studied the photo conversion mechanism of BuSnOOH under radiation.^37^ According to programmed temperature desorption (TPD) results, the C–Sn bond in BnSnOOH was dissociated at 653 K. X-Ray (1486.6 eV) and electron beam (80 eV) could both initiate the detachment of surface organic ligands in the structure, thus inducing the formation of tin oxide structure, resulting in the difference of solubility in the film.

**Fig. 20:**
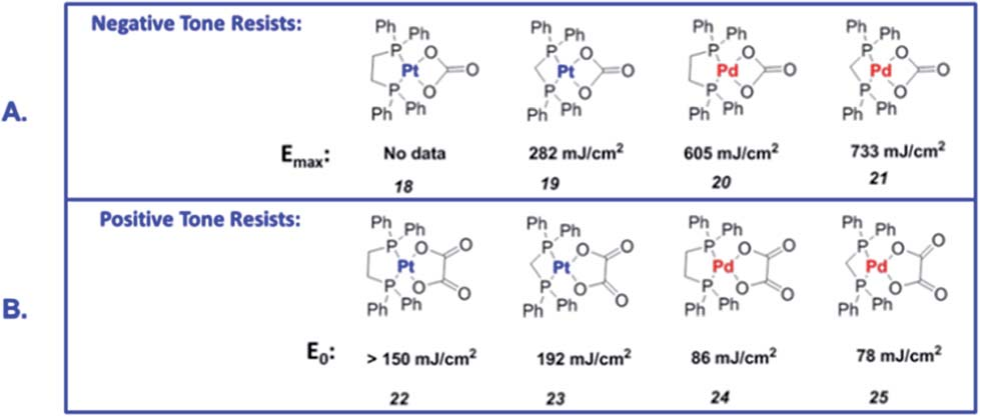
Sensitivity of L_2_M (CO_3_) and L_2_M(C_2_O_4_) to EUV light sources. (A) Pt and Pd carbonates showed negative photoresist properties. (B) Pt and Pd oxalate showed positive photoresist properties. Reprint with permission from ref. 38. Copyright 2015 SIPE.

**Fig. 21:**
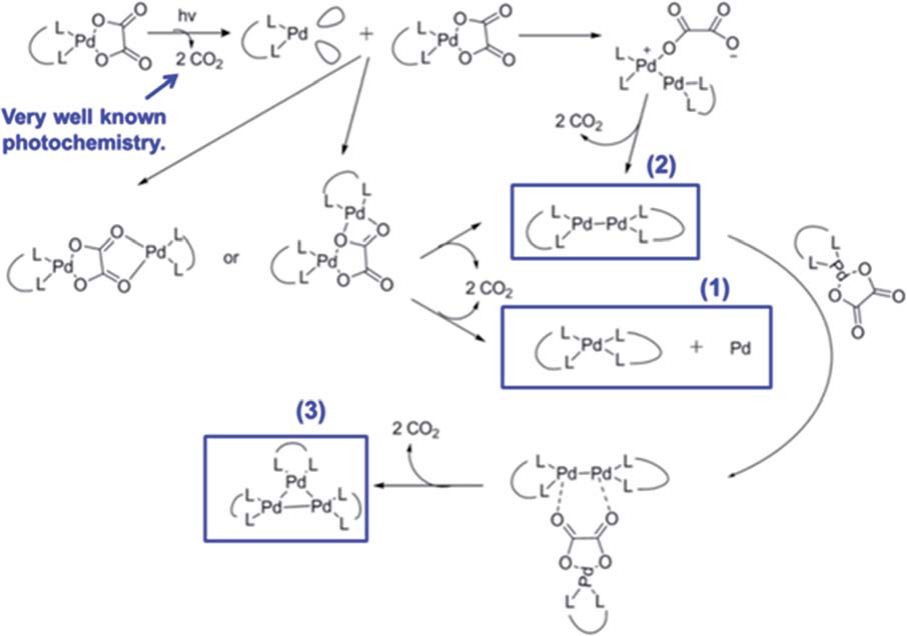
Photoreaction mechanism proposed by the author. Reprint with permission from ref. 38. Copyright 2015 SIPE.

Many Pt and Pd complexes had low sensitivity to EUV, which limited their application in EUV lithography. To solve this problem, Brainard's research team explored the metal carbonates, oxalates (L_2_M(CO_3_) and L_2_M(C_2_O_4_)) of Pt and Pd for the application as EUV photoresists.^38^ Among them, carbonate could be used as negative photoresist, while oxalate was the first reported EUV lithography positive photoresist of mononuclear organometallic compound (developer was a mixed solution of methyl isobutyl ketone and toluene). Fig. 20 showed the relevant metal–organic complex structure, the photoresist^25^ with the best sensitivity could print a line width of 30 nm under the dose of 50 mJ cm^−2^. The author proposed a photo-induced resist conversion mechanism (Fig. 21). After a series of conversion reactions, the metal complex could eliminate polar oxalate and form palladium clusters encapsulated by non-polar ligands to enhance its solubility in organic developer. However, the use of expensive and rare platinum-based resist was not conducive to low-cost mass production, but this research could also help us to further design photoresist with better performance.

## 4. Conclusions and outlook

A variety of inorganic photoresists have been reviewed here. Selective inorganic based photoresists and their lithographic performance have been summarized in Table S1 in ESI.† EUV lithography has a smaller target pattern size. Traditional organic photoresists are difficult to withstand etch conditions, while inorganic photoresists can be promising candidates for this area. For inorganic photoresist, the inorganic core composition generally determines its absorption property towards EUV light source, while the type of organic ligand on the surface determines the photochemical conversion that might occur after exposure. Therefore, from materials view, the design and adjustment of the performance of inorganic photoresist could focus on two factors, one is the composition and structure of core inorganic component and another one is the organic ligand on the surface layer. Inorganic core with ultra small size (1–3 nm) and high uniformity would benefit the resolution and LER of lithography. It is expected that exploration and design of practical synthetic methods to make nanoparticles with precise atomic number and structure is beneficial for developing high-performance photoresist, as it can improve the homogeneity of photoresist film. In addition, changing the surface organic ligand, with different binding strength to the inner inorganic core, could provide a variety of nanomaterials to tune the photochemical reaction that might occur during illumination. Novel types of ligand could be designed to trigger rapid coupling reaction or ligand dissociation reaction upon EUV irradiation and cause the solubility change of nanoclusters. It is expected to obtain new types of inorganic composite photoresists with the optimized trade-off for both resolution, sensitivity and LER.

## Conflicts of interest

There are no conflicts to declare.

## Supplementary Material

RA-010-C9RA08977B-s001
