# Supplementary material for: Review of recent advances in inorganic photoresists
Source: RSC Adv. 2020 Feb 28;10(14):8385–95. doi: 10.1039/c9ra08977b (PMC9049984; doi:10.1039/c9ra08977b)
Supplement: RA-010-C9RA08977B-s001 [file RA-010-C9RA08977B-s001.pdf]

Electronic Supplementary Material (ESI) for RSC Advances.
This journal is © The Royal Society of Chemistry 2020

Review of recent advances in inorganic photoresists

Chaoyun Luo[#,a,b] Chanchan Xu,[#,c] Le Lv,[#,a] Hai Li,[b] Xiaoxi Huang,[*,b] and Wei Liu,[*,b]

a. School of Applied Chemistry and Biological Technology, Shenzhen Polytechnic, 7098 Liuxian Blvd, Nanshan District, Shenzhen, 518055, China.

b. Hoffmann Institute of Advanced Materials, Shenzhen Polytechnic, 7098 Liuxian Blvd, Nanshan District, Shenzhen, 518055, China

c. State-owned Assets Management Office, Shenzhen Polytechnic, 7098 Liuxian Blvd, Nanshan District, Shenzhen, 518055, China.

# These authors contributed equally.

Table S1. Selective examples of inorganic based photoresists and their lithographic performance.[a]

Photoresist Major Composition	Method	Dose	Developer	Positive/Negative	Resolution	Ref
HfO_2	E-beam	103 μC/cm^2	Isopropyl Alcohol	Negative	50 nm line	1
HfO_2	E-beam	--	tert-Amyl Alcohol	Negative	35 nm line	1
HfO_2	E-beam	30 μC/cm^2	Organic Solvent	Negative	40 nm line	2
HfO_2	DUV	--	Organic Solvent	Negative	50 nm h/p line	2
ZrMAA	EUV	5.6 mJ/cm^2	Organic Developer	Negative	32 nm line	3
ZrMAA	EUV	4.2 mJ/cm^2	Organic Developer	Negative	26 nm line	3
$ZircSO_x$	E-beam	999 μC/cm^2	TMAH	Negative	15 nm isolate line	4
$ZircSO_x$	E-beam	810 μC/cm^2	TMAH	Negative	36 nm dense line	4
$[(PhSn)_{12}O_{14}(OH)_6]Cl_2$	EUV	350 mJ/cm^2	66% aqueous isopropanol	Negative	18 nm dense line	5
Tin oxo cage with acetate anion	EUV	68 mJ/cm^2	2/1 isopropanol/H_2O	Negative	30 nm h/p line	6
Inpria Y-Series	EUV	35 mJ/cm^2	a proprietary organic developer	Negative	13 nm h/p line/space	7
3-methyl-phenyl-modified Zn-mTA cluster	EUV	47 mJ/cm^2	Decahydronaphthalene	Negative	15 nm h/p line/space	8
Zn (MA)(TFA)	EUV	27 mJ/cm^2 or 37 mJ/cm^2	Diluted propionic acid in chloroform	Negative	30 nm h/p line/space	9
Tri(phenyl)antimony diacrylate	EUV	5.6 mJ/cm^2	Hexane	Negative	35 nm dense line	10
$(dppm)Pd(C_2O_4)$	EUV	50 mJ/cm^2	20% MIBK/Toluene	Positive	30 nm dense line	11
$[PPh_3(CH_2Ph)][Co(2,2'\text{-bipyridine})(oxalate)_2]$	EUV	30 mJ/cm^2	Methyl ethyl ketone	Negative	20 nm h/p line	12

[a] Abbreviations: MA: methacrylate; MAA: methacrylate acid; TFA: trifluoroacetate; TMAH: tetramethylammonium hydroxide; R: aliphatic or aromatic group; dppm: 1,1-Bis(diphenylphosphino)methane; MIBK: methyl isobutyl ketone.

Reference

1. M. Trikeriotis, W. J. Bae, E. Schwartz, M. Krysak, N. Lafferty, P. Xie, B. Smith, P. A. Zimmerman, C. K. Ober and E. P. Giannelis, *Proc. SPIE*, 2010, **7639**, 76390E.
2. M. Krysak, M. Trikeriotis, E. Schwartz, N. Lafferty, P. Xie, B. Smith, P. Zimmerman, W. Montgomery, E. Giannelis and C. K. Ober, *Proc. SPIE*, 2011, **7972**, 79721C.
3. M. Trikeriotis, M. Krysak, Y. S. Chung, C. Ouyang, B. Cardineau, R. Brainard, C. K. Ober, E. P. Giannelis and K. Cho, *Proc. SPIE*, 2012, **8322**, 83220U.
4. J. Stowers and D. A. Keszler, *Microelectronic Engineering*, 2009, **86**, 730-733.
5. B. Cardineau, R. Del Re, M. Marnell, H. Al-Mashat, M. Vockenhuber, Y. Ekinci, C. Sarma, D. A. Freedman and R. L. Brainard, *Microelectronic Engineering*, 2014, **127**, 44-50.
6. J. Haitjema, Y. Zhang, M. Vockenhuber, D. Kazazis, Y. Ekinci and A. Brouwer, *Journal of Micro/Nanolithography, MEMS, and MOEMS*, 2017, **16**, 033510.

7. A. Grenville, J. T. Anderson, B. L. Clark, P. D. Schepper, J. Edson, M. Greer, K. Jiang, M. Kocsis, S. T. Meyers, J. K. Stowers, A. J. Telecky, D. D. Simone and G. Vandenberghe, *Proc. SPIE*, 2015, **9425**, 94250S.
8. H. Xu, K. Sakai, K. Kasahara, V. Kosma, K. Yang, H. C. Herbol, J. Odent, P. Clancy, E. P. Giannelis and C. K. Ober, *Chemistry of Materials*, 2018, **30**, 4124-4133.
9. N. Thakur, M. Vockenhuber, Y. Ekinci and S. Castellanos, *Proc. SPIE*, 2019, **10957**, 9.
10. J. Passarelli, M. Murphy, R. D. Re, M. M. Sortland, J. Hotalen, L. Dousharm, R. Fallica, Y. Ekinci, M. Neisser, D. A. Freedman and R. L. Brainard, *Journal of Micro/Nanolithography, MEMS, and MOEMS*, 2015, **14**, 1-10, 10.
11. M. Sortland, R. D. Re, J. Passarelli, J. Hotalen, M. Vockenhuber, Y. Ekinci, M. Neisser, D. A. Freedman and R. L. Brainard, *Proc. SPIE*, 2015, **9422**, 942227.
12. M. Wilklow-Marnell, D. Moglia and B. Steimle, *Journal of Micro/Nanolithography, MEMS, and MOEMS*, 2018, **17**, 1.
